# Nitric Oxide Enhances Cytotoxicity of Lead by Modulating the Generation of Reactive Oxygen Species and Is Involved in the Regulation of Pb^2+^ and Ca^2+^ Fluxes in Tobacco BY-2 Cells

**DOI:** 10.3390/plants8100403

**Published:** 2019-10-09

**Authors:** Jiaye Wu, Yue Zhang, Ruizhi Hao, Yuan Cao, Xiaoyi Shan, Yanping Jing

**Affiliations:** 1 College of Biological Sciences and Biotechnology, Beijing Forestry University, Beijing 100083, China; abbywujiaye@126.com (J.W.); yue_zhang@bjfu.edu.cn (Y.Z.); haoruizhizhi@163.com (R.H.); shanxy@bjfu.edu.cn (X.S.); 2 State Key Laboratory of Tree Genetics and Breeding, Chinese Academy of Forestry, Beijing 100091, China; windsor0185@163.com

**Keywords:** NO, Pb^2+^, ROS, flux, Ca^2+^, homeostasis, tobacco BY-2 cell

## Abstract

Lead is a heavy metal known to be toxic to both animals and plants. Nitric oxide (NO) was reported to participate in plant responses to different heavy metal stresses. In this study, we analyzed the function of exogenous and endogenous NO in Pb-induced toxicity in tobacco BY-2 cells, focusing on the role of NO in the generation of reactive oxygen species (ROS) as well as Pb^2+^ and Ca^2+^ fluxes using non-invasive micro-test technology (NMT). Pb treatment induced BY-2 cell death and rapid NO and ROS generation, while NO burst occurred earlier than ROS accumulation. The elimination of NO by 2-4-carboxyphenyl-4,4,5,5-tetramethylimidazoline-1-oxyl-3-oxide (cPTIO) resulted in a decrease of ROS, and the supplementation of NO by sodium nitroprusside (SNP) caused an increased accumulation of ROS. Furthermore, the addition of exogenous NO stimulated Pb^2+^ influx, thus promoting Pb uptake in cells and aggravating Pb-induced toxicity in cells, whereas the removal of endogenous NO produced the opposite effect. Moreover, we also found that both exogenous and endogenous NO enhanced Pb-induced Ca^2+^ effluxes and calcium homeostasis disorder. These results suggest that exogenous and endogenous NO played a critical regulatory role in BY-2 cell death induced by Pb stress by promoting Pb^2+^ influx and accumulation and disturbing calcium homeostasis.

## 1. Introduction

Currently, heavy metal contamination is a major form of environmental pollution owing to emissions from industry, agricultural chemicals, vehicular traffic, and other human activities [1]. Lead is one of the most hazardous metals in the environment and is toxic to plants and other organisms. Like other heavy metals, Pb impairs plants by inhibiting seed germination and plant growth [2,3,4]; reducing nutrient uptake and biomass [5]; disrupting cell membrane permeability, photosynthesis, and cell division [4,6,7]; inhibiting fundamental enzymatic reactions; demolishing cell viability; and inducing cell death [8].

Heavy metals usually result in oxidative stress and a burst of reactive oxygen species (ROS) [9]. In addition to ROS, nitric oxide (NO) may also be rapidly induced in plant cells to regulate plant responses to abiotic stress, including heavy metal toxicity [10,11]. To date, there is a considerable amount of evidence addressing the relationship between ROS and NO signaling in plants. Some studies have reported that exogenous ROS such as H_2_O_2_ could induce the rapid production of NO in plants [12,13]. Others have reported that when exposed to heavy metals, NO has a role in counteracting heavy-metal-induced ROS by scavenging ROS or stimulating the antioxidant defense system of plants [14]. There is also some evidence suggesting that NO-dependent H_2_O_2_ generation and the inhibition of NO synthesis partially prevents a H_2_O_2_ increase under Cd stress [15,16,17]. Groβ et al. found that NO is an important second messenger and can modify ROS signaling or act independently from ROS [18]. Although informative, most of these results come from research focused on cadmium and arsenic, and the related information on Pb stress is still limited.

NO might be supplied with a NO donor to determine the role of exogenous NO on heavy metal tolerance, and it might also be endogenously produced in response to heavy metals. Hence, the dual effect of NO, when exogenously added and endogenously generated, has been documented. Most studies have reported that exogenous NO alleviates metal stress, according to the review of He et al. and Terrón-Camero et al. [19,20]. For example, exogenous NO promotes the recovery of Cd-induced crown root primordia initiation in rice seedlings and partially ameliorates Pb toxicity in wheat roots [21,22]. However, there are also some reports stating that exogenous NO increases heavy metal toxicity. Ma et al. found that exogenous NO promotes Cd^2+^ influxes into BY-2 cells and plays a positive role in CdCl_2_-induced programmed cell death (PCD) [23]. It was also reported that sodium nitroprusside (SNP) pretreatment can increase ROS-mediated Cd cytotoxicity in *Brassica juncea* [24]. Information concerning the role of endogenous NO generated upon heavy metals is still limited, although most studies indicate that endogenous NO contributes to metal toxicity in plants and a few works have demonstrated that endogenous NO counteracts heavy-metal-induced cytotoxicity [14]. Furthermore, an increasing amount of evidence suggests that heavy metal uptake is regulated by endogenous NO, and heavy metal application is often accompanied by a reduction in calcium ions [25,26]. Despite several reports regarding the role and mechanism of NO in heavy metal toxicity and uptake in plants, current knowledge of the role of exogenous and endogenous NO in Pb^2+^ accumulation and Pb toxicity is still limited. Moreover, there is a lack of more direct evidence regarding the function of NO in Pb accumulation and Pb-induced Ca^2+^ variation in plants.

Here, on the basis of addressing cell death in tobacco BY-2 suspension cells exposed to Pb, we investigated the production and the generation sequences of NO and ROS. Then, we analyzed the effect of the NO-donor SNP and the NO-specific scavenger 2-4-carboxyphenyl-4,4,5,5- tetramethylimidazoline-1-oxyl-3-oxide (cPTIO) on NO and ROS generation in relation to Pb-induced cell death. Then, the direction and rate of Pb^2+^ flux across the membrane of tobacco BY-2 cells upon a transient or long exposure to Pb were determined using non-invasive micro-test technology (NMT), and the uptake of Pb was examined by flame atomic absorption spectrometry. In addition, the effects of SNP or cPTIO on Pb-induced Ca^2+^ fluxes were also evaluated. The results presented here show that exogenous NO and Pb-triggered endogenous NO burst contributed to ROS generation in tobacco BY-2 cells, promoted Pb^2+^ influx in cells, and hence increased Pb uptake by the cells, enhanced Pb-induced calcium homeostasis disorder, and played a critical regulatory role in tobacco BY-2 cell death induced by Pb stress.

## 2. Results

### 2.1. Pb Induced PCD in Tobacco BY-2 Cells

**Figure 1:**
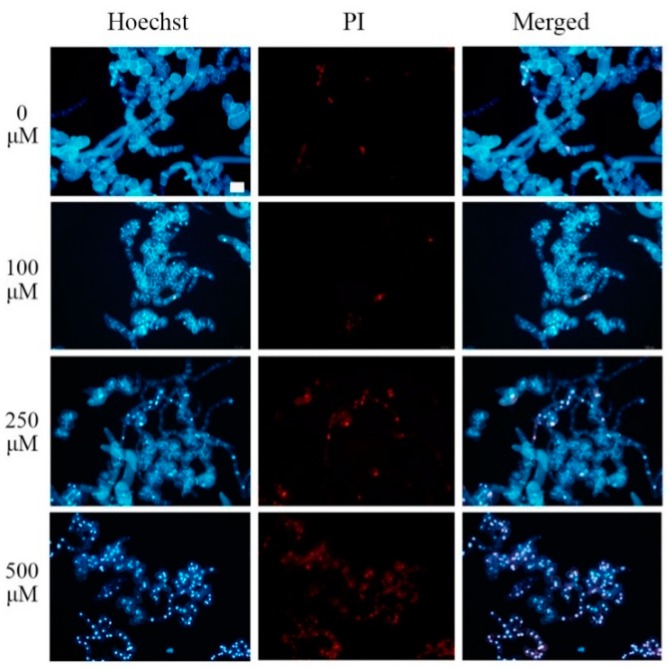
Tobacco BY-2 cells double stained with Hoechst 33342 and propidium iodide (PI). Hoechst 33342 and PI double staining in cultured tobacco BY-2 cells treated with different concentrations (0, 100, 250, and 500 μM) of Pb(NO_3_)_2_ for 24 h. Scale bar = 100 μm.

BY-2 suspension cells were treated with 0, 100, 250, and 500 μM Pb(NO_3_)_2_ for 24 h, and their nuclear morphology was observed after staining with 5 μg/mL Hoechst 33342. The dead cells were determined by 3 μg/mL propidium iodide (PI), which is generally excluded from viable cells for its membrane-impermeable properties. As shown in Figure 1, most control cells were Hoechst positive and emitted blue fluorescence, whereas the dead cells which emitted red fluorescence were hardly found. The proportion of PI-positive cells increased obviously with the increase of the concentration of Pb(NO_3_)_2_. Among the Hoechst-positive cells, the nucleus in some cells was round and uniformly stained, whereas in other cells appeared stretched and granular staining (Figure S1, Supplementary Materials), implying that these cells were undergoing PCD. 

Next, we examined the internucleosomal fragmentation of DNA triggered by endonucleases using a terminal deoxynucleotidyl transferase-mediated nick end labeling (TUNEL) assay. After 24 h of treatment with 100 and 250 μM Pb(NO_3_)_2_, the nuclei appeared TUNEL positive (Figure S2, Supplementary Materials). Conversely, almost all of the nuclei in the control cells and 500 μM Pb(NO_3_)_2_-treated cells were TUNEL negative. Positive and negative controls were included and further confirmed the absence of artifacts.

### 2.2. Pb Triggered ROS and NO Bursts in Tobacco BY-2 Cells

**Figure 2:**
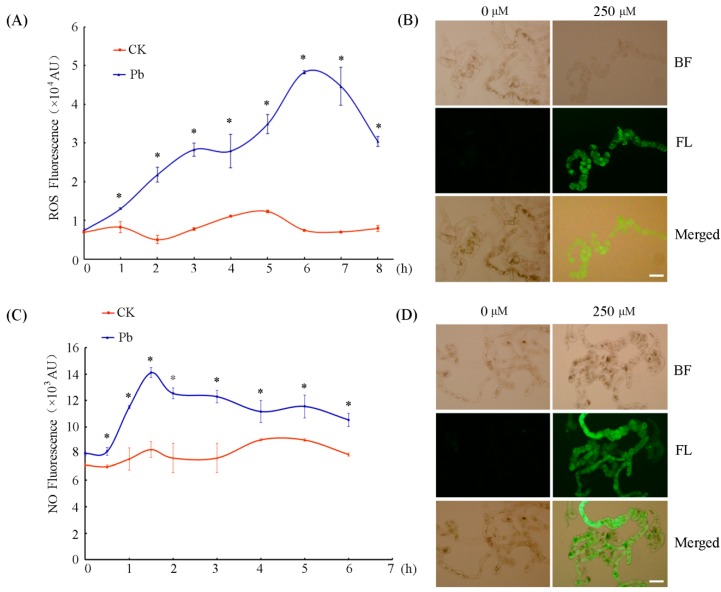
ROS and NO released in tobacco BY-2 cells treated with 250 μM Pb(NO_3_)_2_. (**A**) ROS production was measured at different times after treatment with or without 250 μM Pb(NO_3_)_2_. (**B**) The distribution of ROS in BY-2 cells detected with 2’,7’-dichlorofluorescin diacetate (DCFH-DA) after 6 h of treatment with 250 μM Pb(NO_3_)_2_. Cells that received the same volume of distilled water were used as a control. Scale bar = 100 μm. (**C**) NO release was measured at different times after treatment with or without 250 μM Pb(NO_3_)_2_. (**D**) The distribution of NO in BY-2 cells detected with 3-amino,4-aminomethyl-2’,7’-difluorescein diacetate (DAF-FM DA) after 1.5 h of treatment with 250 μM Pb(NO_3_)_2_. Cells that received the same volume of distilled water were used as a control. Scale bar = 100 μm. Each value in A and C represents the average of three independent experiments and the bars indicate the standard error of the mean. Asterisks indicate values that are significantly different from those of control cells (*p* < 0.05). CK, Control; BF, Bright Field; FL, Fluorescence.

Both ROS and NO are often produced in large amounts during plant response to various stresses and play key roles in plant PCD during development and defense [17,27,28,29]. The influence of Pb on ROS and NO production in tobacco BY-2 cells was examined in this work. As shown in Figure 2, the ROS contents in the Pb-treated cells presented a time-dependent increase after Pb treatment and reached the highest peak at about 6 h after Pb exposure (Figure 2A,B). Measurements of NO released in the cells revealed an immediate increase right after Pb treatment in comparison with that in control cells. Following the treatment with 250 μM Pb(NO_3_)_2_, the release of NO increased rapidly. At 1.5 h, the NO reached the highest level, which was about 1.70-fold higher than that in control cells (Figure 2C,D). These results suggest that the NO burst occurred earlier than that of ROS under Pb stress in tobacco BY-2 cells.

### 2.3. NO Contributed to Pb-Induced ROS Production in Tobacco BY-2 Cells

**Figure 3:**
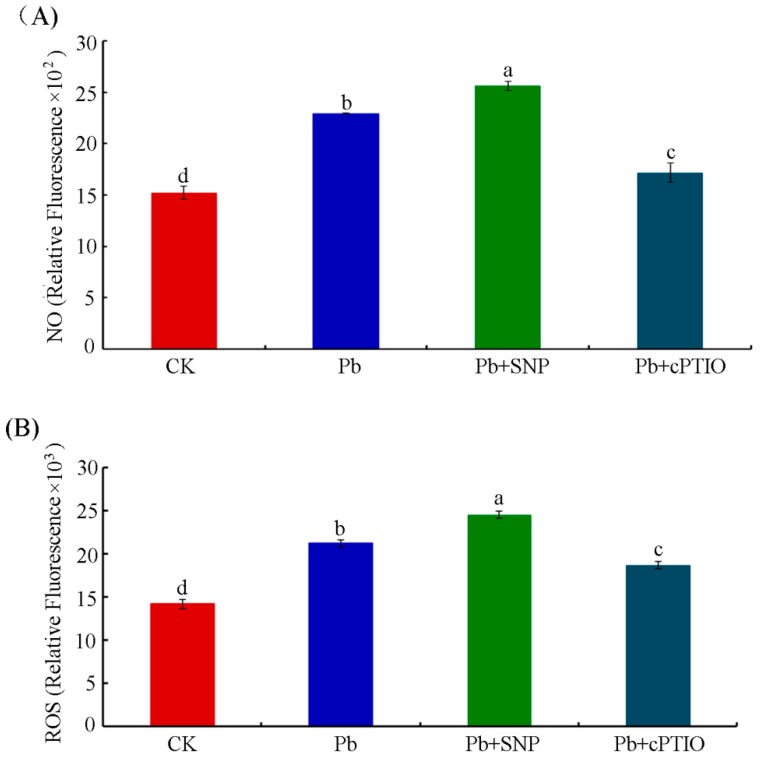
Effects of SNP or cPTIO on NO or ROS levels in tobacco BY-2 cells treated with 250 μM Pb(NO_3_)_2_. (**A**) NO content was measured 1.5 h after Pb treatment. (**B**) ROS levels were detected 6 h after Pb treatment. Bars with different lowercase letters in each panel are significantly different (*p* < 0.05). CK, Control.

The relationship of ROS and NO signaling in plants has been extensively studied, showing that NO may be an upstream signaling molecule for H_2_O_2_ in the auxin signal transduction pathway during adventitious root development in marigold [30]. There are also reports that H_2_O_2_ leads to quick NO production in guard cells of *Phaseolus aureus* [31]. In this work, Pb induced both ROS and NO production in tobacco BY-2 cells. Considering that the Pb-induced NO peak occurred in advance of the ROS peak, the NO-donor SNP and the NO-specific scavenger cPTIO were used to investigate the possible role of NO in ROS production. According to the emerging time of Pb-induced NO and ROS peaks, we treated tobacco BY-2 cells with 250 μM Pb(NO_3_)_2_ in combination with 0.5 μM SNP or 100 μM cPTIO for 6 h and 1.5 h, respectively, after which cells were detected for ROS and NO levels. As shown in Figure 3, compared with the control, Pb treatment resulted in obviously more NO and ROS released in tobacco BY-2 cells. When the exogenous NO-donor SNP was applied to Pb-treated cells, it resulted in a notable increase in the NO content, whereas the NO levels of the cells treated with cPTIO together with Pb were markedly decreased compared with those of the cells treated with Pb alone (Figure 3A). Meanwhile, the presence of SNP markedly increased Pb-induced ROS production. In contrast, cPTIO could reverse, in part, the endogenous levels of ROS induced by Pb (Figure 3B).

**Figure 4:**
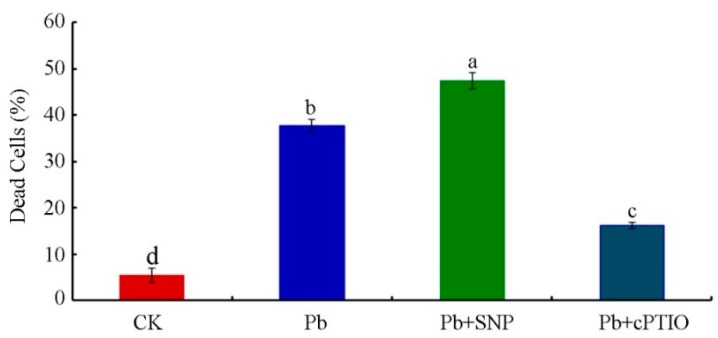
Effects of SNP or cPTIO on cell viability in tobacco BY-2 cells treated with 250 μM Pb(NO_3_)_2_ for 24 h. Bars with different lowercase letters in each panel are significantly different (*p* < 0.05). CK, Control.

We further analyzed the effect of SNP and cPTIO on cell viability in tobacco BY-2 cells treated with 250 μM Pb(NO_3_)_2_ for 24 h. As shown in Figure 4, at 24 h, the portion of dead cells increased from 37.83% to 47.45% in the presence of 0.5 μM SNP, which was about 1.25-fold higher than that under Pb stress alone. Meanwhile, the number of dead cells was reduced by cPTIO from 37.83% to 16.21% after treatment with 250 μM Pb(NO_3_)_2_ for 24 h. The results suggest that NO played a key role in Pb-induced ROS production and, subsequently, cell death.

### 2.4. NO Increased Pb^2+^ Influx in Tobacco BY-2 Cells

**Figure 5:**
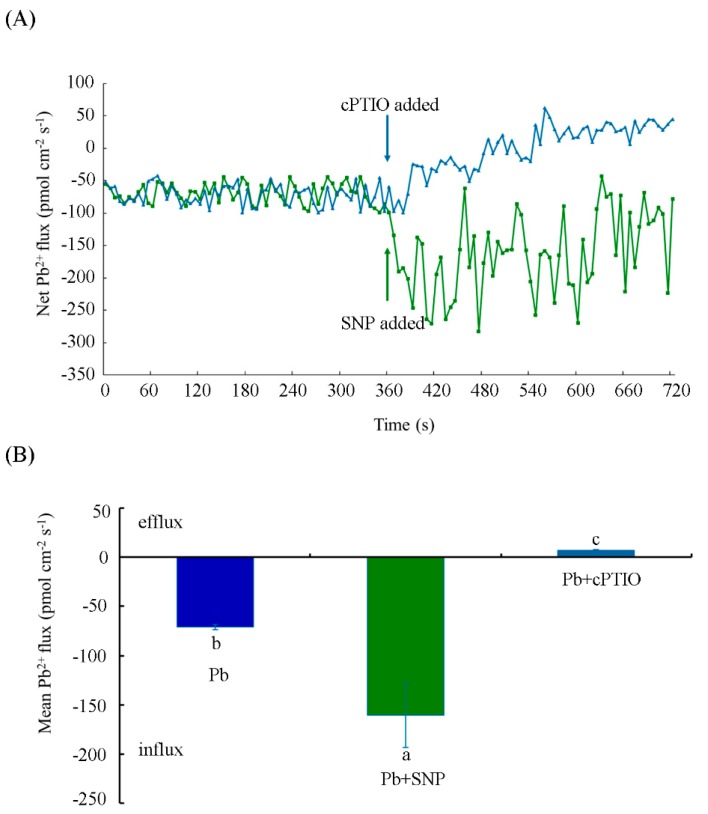
Influence of NO on Pb^2+^ fluxes in tobacco BY-2 cells before and after applications of SNP and cPTIO under Pb stress. (**A**) Net Pb^2+^ fluxes in tobacco BY-2 cells incubated with 250 μM Pb(NO_3_)_2_ and then 0.5 μM SNP (or 100 μM cPTIO) applied at 360 s to investigate the fluctuation of Pb^2+^ flux. (**B**) The mean rate of Pb^2+^ fluxes in tobacco BY-2 cells treated with 250 μM Pb(NO_3_)_2_ in the absence or presence of 0.5 μM SNP or 100 μM cPTIO. Different lowercase letters show significant difference (*p* < 0.05).

NMT is a promising technique for investigating the transfer of metal ions in certain regions of plants and organisms [32,33]. In this work, four-day-old tobacco BY-2 cells were incubated with 250 μM Pb(NO_3_)_2_ and Pb^2+^ flux was immediately measured by NMT. A constant net Pb^2+^ influx with a mean value of 70.40 ± 2.70 pmol cm^−2^s^−1^ was detected after exposure to 250 μM Pb(NO_3_)_2_ (Figure 5A). After the addition of 0.5 μM SNP, the Pb^2+^ influx was significantly increased and reached a rate of 160.56 ± 32.83 pmol cm^−2^s^−1^, with a significant increase of 128.06%. In contrast, treatment with 100 μM cPTIO significantly inhibited the Pb^2+^ influx in comparison with Pb treatment alone, and even a slight net Pb^2+^ efflux of 6.75 ± 0.85 pmol cm^−2^s^−1^ was observed (Figure 5A,B). These results suggest that the Pb^2+^ flux significantly changed in the presence of SNP or cPTIO within a short exposure time to Pb(NO_3_)_2_.

**Figure 6:**
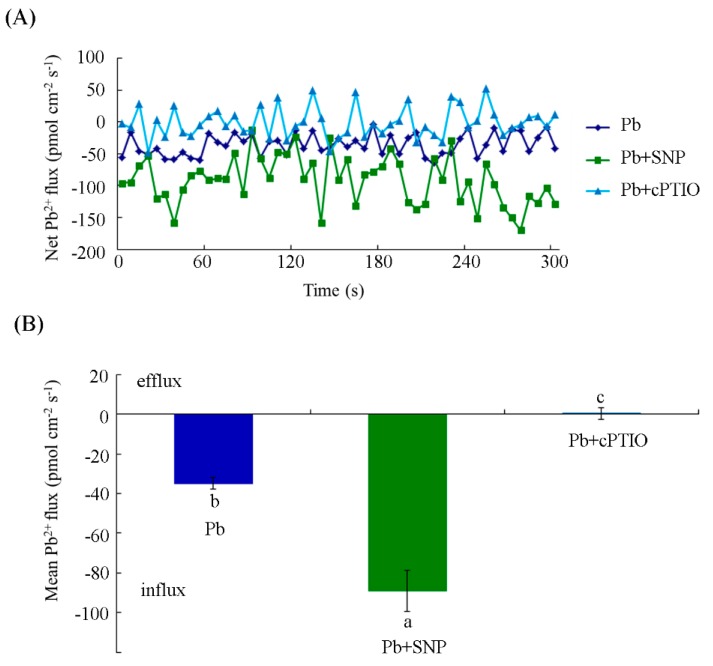
Effects of NO on Pb^2+^ fluxes in tobacco BY-2 cells after different treatments for 10 h. (**A**) Net Pb^2+^ fluxes in tobacco BY-2 cells incubated with 250 μM Pb(NO_3_)_2_ alone, with 0.5 μM SNP, or with 100 μM cPTIO. (**B**) The mean rate of Pb^2+^ fluxes in tobacco BY-2 cells treated with 250 μM Pb(NO_3_)_2_ alone, with 0.5 μM SNP, or with 100 μM cPTIO. Different lowercase letters show significant difference (*p* < 0.05).

We also measured the Pb^2+^ flux in cells with different treatments for a long period of time. Here, 10 h treatment rather than 24 h was chosen, considering that the detection of ion fluxes across the membrane requires viable cells. As shown in Figure 6, a net Pb^2+^ influx into the tobacco BY-2 cells, the mean value of which was 34.94 ± 2.98 pmol cm^−2^s^−1^, was found at 10 h under Pb treatment alone. Upon addition of SNP, the average Pb^2+^ flux increased remarkably to 88.98 ± 10.38 pmol cm^−2^s^−1^. The mean value of Pb^2+^ flux in the presence of SNP was about 2.55-fold higher than that in the control cells treated with Pb alone. However, cells exposed to cPTIO exhibited minimal Pb^2+^ efflux, with a mean value of 0.37 ± 2.83 pmol cm^−2^s^−1^. The above results indicate that NO enhanced Pb^2+^ influx into the cells at 10 h. 

### 2.5. NO Promoted Pb Uptake to Aggravate Pb Toxicity

**Figure 7:**
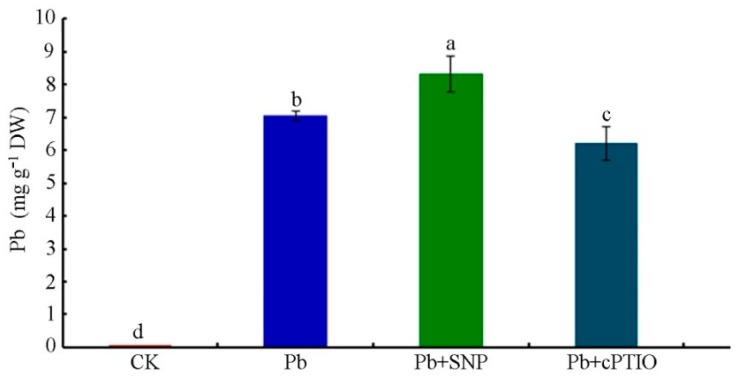
Influence of SNP and cPTIO on Pb uptake by tobacco BY-2 cells. Four-day-old tobacco BY-2 cells treated with 250 μM Pb(NO_3_)_2_ alone, with 0.5 μM SNP, or with 100 μM cPTIO, respectively, were harvested 24 h later for determination of Pb. Cells that received the same volume of distilled water were used as a control. Data are means ± SD of three replicates. Different lowercase letters show significant differences (*p* < 0.05) between the means. CK, Control.

In order to determine the role of NO in Pb uptake, we investigated the effects of the NO-donor SNP and NO-specific scavenger cPTIO on the Pb content in tobacco BY-2 cells exposed to Pb. As shown in Figure 7, the Pb content of cells in the presence of 0.5 μM SNP significantly increased to 8.33 ± 0.55 mg g^−1^ DW, whereas it was 7.04 ± 0.13 mg g^−1^ DW in cells treated with Pb alone. It was about 18.3% higher with the addition of SNP. In contrast, the Pb content was remarkably reduced to 5.00 ± 0.22 mg g^−1^ DW when supplied with 100 μM cPTIO versus Pb-treated cells alone. Application of cPTIO reduced the Pb content in tobacco BY-2 cells by 28.9% as compared with Pb-stressed cells.

### 2.6. NO Enhanced Pb-Induced Calcium Homeostasis Disorder

**Figure 8:**
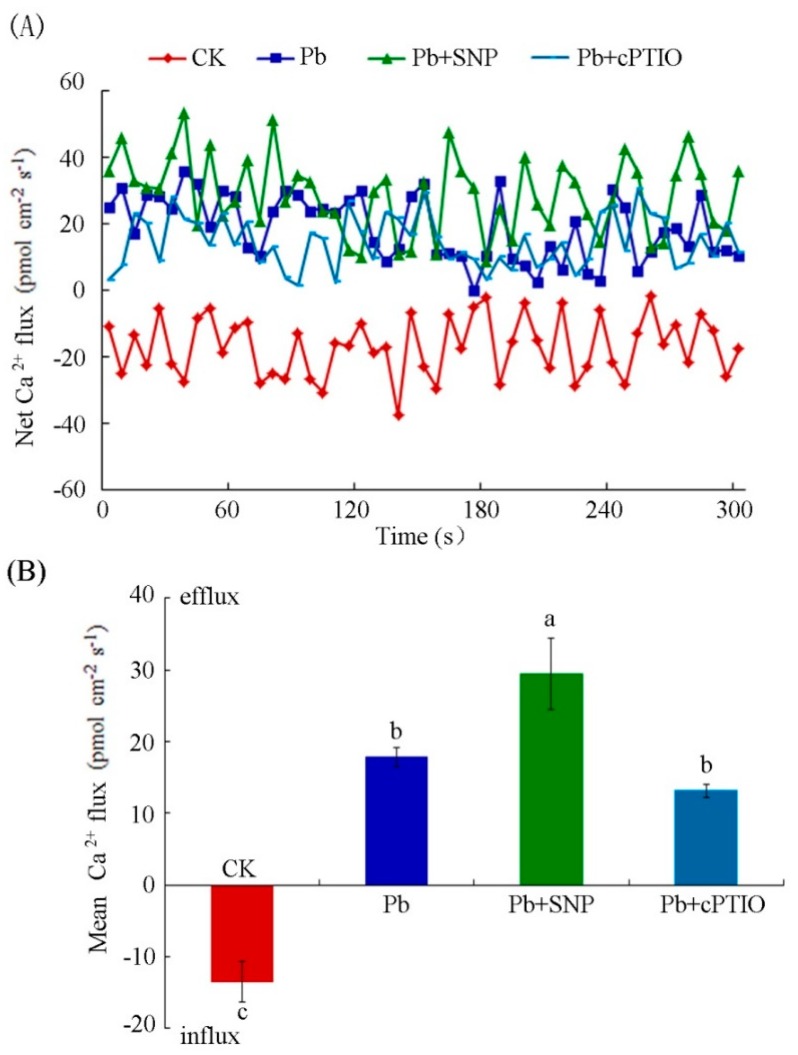
Effects of NO on Ca^2+^ fluxes in tobacco BY-2 cells after different treatments for 10 h. (**A**) Net Ca^2+^ fluxes in tobacco BY-2 cells treated with 250 μM Pb(NO_3_)_2_ alone, with 0.5 μM SNP, or with 100 μM cPTIO. (**B**) The mean rate of Ca^2+^ fluxes in tobacco BY-2 cells treated with 250 μM Pb(NO_3_)_2_ alone, with 0.5 μM SNP, or with 100 μM cPTIO. Cells that received the same volume of distilled water were used as a control. Different lowercase letters show significant difference (*p* < 0.05). CK, Control.

Calcium, as nutrition and signal molecule, plays an important function in various life activities of plants. It is generally considered to alleviate heavy metal toxicities. Previous studies have found that Pb blocks calcium absorption in plants, thus producing toxic effects on plant growth [34]. In this work, NO promoted Pb^2+^ influx and participated in Pb uptake by BY-2 suspension cells. We further detected the effect of NO on Pb-induced changes of Ca^2+^ fluxes. As shown in Figure 8, a net Ca^2+^ influx into tobacco BY-2 cells, the mean value of which was 13.54 ± 2.78 pmol cm^−2^s^−1^, was detected under control cells. Upon Pb stress, the average Ca^2+^ influx was suppressed and the pattern of Ca^2+^ influx changed to a Ca^2+^ efflux, with a mean value of 17.88 ± 1.33 pmol cm^−2^s^−1^. Tobacco BY-2 cells exposed to 0.5 μM SNP exhibited a significantly elevated Ca^2+^ efflux (29.42 ± 4.97 pmol cm^−2^s^−1^) compared with that of the cells treated with Pb alone. The Ca^2+^ efflux was decreased to a mean value of 13.18 ± 0.91 pmol cm^−2^s^−1^ in the presence of 100 μM cPTIO in comparison with that with Pb treatment alone. However, this effect was not significant. These data suggest that NO enhanced Pb-induced calcium homeostasis disorder in tobacco BY-2 cells.

## 3. Discussion

Environmental pollution with toxic heavy metals poses a rising threat to both the ecosystem and human health [35]. Heavy metals also impose harmful effects on plant growth and metabolism. Among various heavy metals, lead is one of the most toxic and frequently faced contaminants owing to its toxic potential to plants and other organisms as well as its global-scale distribution [36,37]. Exposure to Pb stress causes damage to the chloroplast ultrastructure [38], disturbance of nutrient metabolism [39], inhibition of plant growth and photosynthesis [7,40], suppression of cell division [4], and, consequently, cell death [8]. In this study, different concentrations of Pb stress on tobacco BY-2 cells resulted in different degrees of cell death (Figure 1). Furthermore, chromatin condensation and granular staining nuclei, which are considered the hallmark of PCD, were found in tobacco BY-2 suspension cells treated with 250 μM Pb(NO_3_)_2_ (Figure S1, Supplementary Materials). In addition, DNA strand breaks were detected by the TUNEL assay in cells treated with 250 μM Pb(NO_3_)_2_ (Figure S2, Supplementary Materials). Based on these morphological changes, these data confirmed that Pb leads to PCD in tobacco BY-2 cells, which is consistent with previous reports [41].

The toxicity of heavy metals quite often evokes the generation of ROS, which might react with many cellular organelles to cause cell damage [42]. It was reported that 0.5–1 mM Pb significantly induced cell death in rice root cells by triggering ROS production [43]. Besides ROS, NO (a bioactive molecule) has also been found to be a crucial messenger molecule in plant response to heavy metals [44,45,46]. The results presented in this work show that the production of ROS and NO increased dramatically in tobacco BY-2 cells treated with 250 µM Pb(NO_3_)_2_. However, the peak of NO (at 1.5 h) appeared much earlier than that of ROS (at 6 h) (Figure 2). The levels of ROS and NO have been reported to be reciprocally controlled or affected by each other [47]. For example, it has been found that exogenous ROS such as H_2_O_2_ induce NO generation in *Hypericum perforatum* cell cultures and *Phaseolus aureus* guard cells [13,31]. Meanwhile, some reports have demonstrated that NO provides protection as an antioxidant by scavenging active oxygen species generated by Cd^2+^ stress in sunflower leaves [10] and wheat roots [46], while other reports have demonstrated that heavy-metal-induced NO production promotes ROS accumulation in the root of *Solanum nigrum* [48]. In the present study, Pb-induced NO reached peaks at about 1.5 h. However, ROS accumulation occurred at about 6 h. The time course suggests that NO might act upstream of ROS in tobacco BY-2 cell responses to Pb stress. We used the NO-donor SNP and the NO-specific scavenger cPTIO to investigate the role that NO plays in the Pb-induced generation of ROS. The NO-specific scavenger cPTIO not only diminished the NO content but also decreased the production of ROS. Accordingly, when exogenous NO was supplied by SNP, besides NO, ROS levels were also notably raised (Figure 3). Recently, it has been reported that stress-induced ROS generation in plants is modulated through NO crosstalk with ROS-scavenging enzymes, thereby modulating ROS status [49]. Kaur et al. reported that ROS generation decreased upon exogenous NO addition when wheat roots were treated with 50 and 250 μM Pb. It is attributed to the role of NO directly scavenging ROS as an antioxidant [22]. NO displays both antioxidant and pro-oxidant activity which is determined by the time and location of NO production, and the quantity of NO generated in cells. Here, under the same concentration of Pb stress, our study led to the opposite conclusion, that is, both exogenous and endogenous NO promote ROS generation in tobacco BY-2 cells upon Pb stress. The reason for the controversy may be attributed to the different plant species used as well as the different content of exogenous NO supplied. 

It has been documented that NO and ROS could influence one another, and the interactions between them might be the real cause of cell death in plants [27]. In this study, we also analyzed the effect of exogenous and endogenous NO on the cell viability of BY-2 cells. We found that the addition of exogenous NO significantly enhanced Pb-induced cell death, whereas the removal of endogenous NO alleviated Pb-induced cell death compared with Pb treatment alone (Figure 4). Hence, our study indicates that both exogenous and endogenous NO enhanced Pb toxicity in tobacco BY-2 cells. Most reports indicate that exogenous NO supplementation has a role in the protection of plants by alleviating heavy metal stress, including Cd [50], Cu [51], Pb [52], and so forth. There are a few reports showing that the application of exogenous NO in combination with heavy metals enhances metal toxicity [53]. The contribution of endogenous NO to plant metal stress was also reported to exert both cytotoxic and cytoprotective effects [14]. The reasons for this discrepancy can be probably due to the variety of the plant tissues used, the age of the plants, the concentrations and the duration of heavy metal exposure, and so forth.

NMT has been reported to be an effective approach to studying ion uptake and accumulation in plants and animals. The fluxes of ions, such as Cd^2+^, Ca^2+^, K^+^, Pb^2+^, and so forth, can be measured by NMT under normal physiological conditions [31,53,54]. Heavy metal transport is crucial for understanding metal uptake mechanisms in plants. In this study, we used NMT to show that a constant net Pb^2+^ influx occurred in tobacco BY-2 cells under short- and long-term Pb treatment. Exogenous NO supplied with SNP increased the Pb^2+^ influx, whereas the removal of NO by cPTIO resulted in a slight efflux of Pb^2^^+^ (Figure 5 and Figure 6). Moreover, our data on Pb content determination indicate that exogenous NO and Pb-induced endogenous NO promote Pb accumulation in tobacco BY-2 cells (Figure 7), which is consistent with our results of Pb^2+^ fluxes determined by NMT. The results support a previous study showing that the Pb-induced production of NO plays a critical role in Pb uptake by *Pogonatherum crinitum* root cells [11]. The promotion of Pb uptake by exogenous and endogenous NO also implicates the enhancement of NO on Pb toxicity in BY-2 cells. Moreover, it was reported that Pb accumulated in plants reduced calcium uptake [55]. Using NMT to determine whether Pb alters the pattern of Ca^2+^ flux across the membrane and to investigate the role of NO during this process, we also measured Ca^2+^ flux upon Pb stress supplied with or without SNP and cPTIO. Our results indicate that Pb stress obviously induced Ca^2+^ efflux from cells, and NO acted positively during this course (Figure 8). Thus, NO induced Pb^2+^ influx and enhanced Pb-induced calcium homeostasis disorder.

## 4. Materials and Methods 

### 4.1. Cell Culture

Tobacco BY-2 cells were cultured in MS medium containing 30 g L^−1^ sucrose and 1 mg L^−1^ 2,4-D (pH 5.8). The cells were grown in darkness at 25 ± 2 °C on a rotary shaker at 110 rpm and subcultured at a dilution of 1:10 per week. 

### 4.2. Hoechst and PI Double Staining

Cell activity and nuclear morphology were detected using the Hoechst and PI double staining method [23]. Four-day-old tobacco BY-2 cells were treated under different conditions for 24 h. The cells were harvested by centrifugation at 1000× *g* for 2 min, washed twice with fresh medium, and then resuspended in 300 μL of assaying buffer containing the Hoechst 33342 and PI fluorescence (Beyotime, Jiangsu, China) dye for 30 min at room temperature. Next, the stained cells were washed twice with 0.1 M PBS (pH 7.4) and resuspended. The cells were observed with a fluorescence microscope (Olympus BX61, Tokyo, Japan) with an excitation filter of 330–385 nm. For each sample, five different nonoverlapping microscope fields, each containing at least 100 cells, were randomly chosen. Cell death was calculated as the percentage of dead cells to the total number of cells. All data are presented as the means ± SD of three replicates from three independent experiments.

### 4.3. Detection of NO and ROS Production 

The generation of NO and ROS in tobacco BY-2 cells was investigated using the fluorescent dyes DAF-FM DA (3-amino,4-aminomethyl-2’,7’-difluorescein diacetate) and DCFH-DA (2’,7’-dichlorofluorescin diacetate) (Beyotime, Jiangsu, China), respectively. Briefly, tobacco BY-2 cells were cultured for four days and then treated with 250 μM Pb(NO_3_)_2_ in the presence or absence of 100 μM cPTIO or 0.5 μM SNP (Sigma-Aldrich, St. Louis, MO, USA). Next, cells were loaded with 20 μM DAF-FM DA or 20 μM DCFH-DA for 30 min at 37 °C in the dark and then washed three times in fresh PBS (pH 7.4). The fluorescence was detected by a microplate reader (Tecan Infinite M200, Männedorf, Switzerland) with an excitation of 490 nm for NO and 488 nm for ROS and an emission of 520 nm for NO and 525 nm for ROS. Cells treated with the same volume of distilled water (0 μM Pb(NO_3_)_2_) were used as a control. All data are presented as the means ± SD of three replicates from three independent experiments. In addition, after treated with 250 μM Pb(NO_3_)_2_ for 6 h and 1.5 h, respectively, the highest accumulation of ROS and NO in the BY-2 cells was detected under an Olympus BX61 fluorescence microscope at an excitation wave length of 460–480 nm.

### 4.4. Determination of Pb Content

Four-day-old tobacco BY-2 cells were treated with 250 μM Pb(NO_3_)_2_ in the presence or absence of 0.5 μM SNP or 100 μM cPTIO (Sigma-Aldrich, St. Louis, MO, USA) for 24 h. Collected cells were dried for 12 h at 70 °C and then digested with a mixture of HNO_3_/HClO_4_ (5:1, v:v). The Pb content was determined by a flame atomic absorption spectrometer (Shimadzu AA-7000, Kyoto, Japan). All data are presented as the means ± SD of three replicates from three independent experiments.

### 4.5. Measurement of Pb^2+^ and Ca^2+^ Fluxes 

Both Pb^2+^ and Ca^2+^ fluxes were investigated by using NMT (NMT100 Series, Younger USA LLC, Amherst, MA, USA) at Xuyue (Beijing) Sci. & Tech. Co., Ltd., Beijing, China. Four-day-old tobacco BY-2 cells were prepared according to the method described by Ma et al [23] and then transferred to a measuring chamber containing 3 mL of measuring solution for Pb^2+^ (0.1 mM KCl, 0.05 mM CaCl_2_, 0.05 mM MgCl_2_, 0.5 mM NaCl, 0.25 mM Pb(NO_3_)_2_, 0.3 mM Mes, and 3% sucrose; pH 5.8) in the presence of 250 μM Pb(NO_3_)_2_. To measure the Pb^2+^ flux at the initial start time, cells that showed stable fluctuations in the preliminary detection were chosen for the subsequent net Pb^2+^ flux measurements with SNP or cPTIO. Briefly, the SNP or cPTIO stock solution was slowly added to the measuring solution until the final concentration reached 0.5 or 100 μM. Then, the recording of flux was restarted and continued for a further period of 5–10 min. Furthermore, the mean values for different treatments were determined from at least six cells to illustrate Pb^2+^ flux variations upon different pharmacological applications. Cells incubated in standard medium with 250 μM Pb(NO_3_)_2_, 250 μM Pb(NO_3_)_2_, and 0.5 μM SNP, or 250 μM Pb(NO_3_)_2_ and 100 μM cPTIO for 10 h were also collected for Pb^2+^ and Ca^2+^ flux measurements. The measuring solution for Ca^2+^ fluxes included 0.1 mM KCl, 0.05 mM CaCl_2_, 0.05 mM MgCl_2_, 0.5 mM NaCl, 0.3 mM Mes, and 3% sucrose (pH 5.8). The data obtained were converted into specific ion influx values (pmol cm^−2^s^−1^) as described before [56]. At least six cells were used to measure the Pb^2+^ or Ca^2+^ fluxes in each treatment.

### 4.6. Statistical Analysis

The data were analyzed using a one-way analysis of variance (ANOVA) and significant differences among the experimental data were set to *p* = 0.05.

## 5. Conclusions

**Figure 9:**
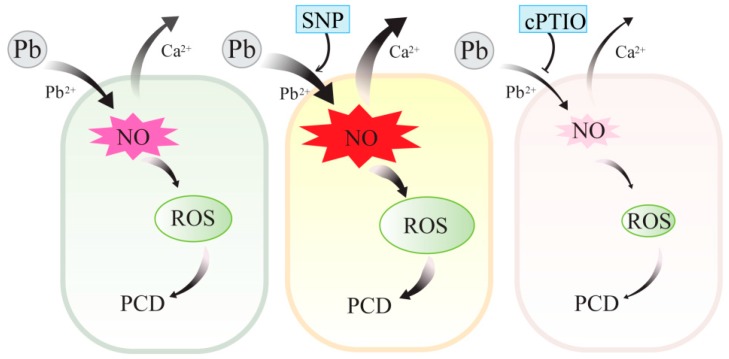
A schematic graphic of NO function in enhancing cytotoxicity of Pb by modulating the generation of ROS, promoting Pb^2+^ influx into the cells, and disturbing the Ca^2+^ homeostasis.

In conclusion, as shown in the schematic graphic (Figure 9), we showed that Pb stress induced Pb^2+^ influx and the generation of ROS and NO. Exogenous and endogenous NO induced by Pb stress acted upstream of ROS and promoted the accumulation of ROS and subsequent cell death in tobacco BY-2 cells. Both exogenous and endogenous NO enhanced Pb toxicity in tobacco BY-2 cells, and the mechanism may attribute to the ability of NO to stimulate Pb^2+^ influx and thus promote Pb uptake and aggravate Pb-induced Ca^2+^ homeostasis disorder in BY-2 cells. These findings lead to a better understanding of the mechanism of NO underlying Pb cytotoxicity in plant cells.

## Supplementary Materials

The following are available online at https://www.mdpi.com/2223-7747/8/10/403/s1, Method: TUNEL assay, Figure S1: Typical cells with nuclei containing condensed or granular chromatin. Hoechst 33342 staining in cultured tobacco BY-2 cells treated with 250 μM Pb(NO_3_)_2_ for 24 h. Scale bar = 50 μm, Figure S2: Programmed cell death detection using the TUNEL assay. Tobacco BY-2 cells that received the same volume of distilled water were used as a control. Four-day-old tobacco BY-2 cells were treated with different concentrations (100, 250, and 500 μM) of Pb(NO_3_)_2_ for 24 h. Left column: TUNEL images; middle column: PI images; right column: merged images of TUNEL and PI. Scale bar = 100 μm.

## References

[1] Malizia D., Giuliano A., Ortaggi G., Masotti A. (2012). Common plants as alternative analytical tools to monitor heavy metals in soil. Chem. Cent. J..

[2] Mesmar-Mn J.K. (1991). The toxic effect of lead on seed germination, growth, chlorophyll and protein contents of wheat and lens. Acta Biol. Hung..

[3] Liu D., Zou J., Meng Q., Zou J., Jiang W. (2009). Uptake and accumulation and oxidative stress in garlic (*Allium sativum* L.) under lead phytotoxicity. Ecotoxicology.

[4] Kozhevnikova A.D., Seregin I.V., Bystrova E.I., Belyaeva A.I., Kataeva M.N., Ivanov V.B. (2009). The effects of lead, nickel, and strontium nitrates on cell division and elongation in maize roots. Russ. J. Plant Physiol..

[5] Nareshkumar A., Krishnappa B.V., Kirankumar T.V., Kiranmai K., Lokesh U., Sudhakarbabu O., Sudhakar C. (2014). Effect of Pb-stress on growth and mineral status of two groundnut (*Arachis hypogaea* L.) cultivars. J. Plant Sci..

[6] Gupta D., Nicoloso F., Schetinger M., Rossato L., Pereira L., Castro G., Srivastava S., Tripathi R. (2009). Antioxidant defense mechanism in hydroponically grown *Zea mays* seedlings under moderate lead stress. J. Hazard. Mater..

[7] Ling Q., Hong F.S. (2009). Effects of Pb^2+^ on the structure and function of photosystem II of *Spirodela polyrrhiza*. Biol. Trace. Elem. Res..

[8] Huang T.L., Huang H.J. (2008). ROS and CDPK-like kinase-mediated activation of MAP kinase in rice roots exposed to lead. Chemosphere.

[9] Corpas F.J., Barroso J.B. (2017). Lead-Induced stress, which triggers the production of nitric oxide (NO) and superoxide anion (O_2_^−^) in *Arabidopsis* peroxisomes, affects catalase activity. Nitric Oxide.

[10] Laspina N.V., Groppa M.D., Tomaro M.L., Benavides M.P. (2005). Nitric oxide protects sunflower leaves against Cd-induced oxidative stress. Plant Sci..

[11] Yu Q., Sun L., Jin H., Chen Q., Chen Z., Xu M. (2012). Lead-induced nitric oxide generation plays a critical role in lead uptake by *Pogonatherum crinitum* root cells. Plant Cell Physiol..

[12] Wang Y., Ries A., Wu K., Yang A., Crawford N.M. (2010). The Arabidopsis prohibitin gene phb3 functions in nitric oxide–mediated responses and in hydrogen peroxide–induced nitric oxide accumulation. Plant Cell.

[13] Xu M.J., Dong J.F., Zhang X.B. (2008). Signal interaction between nitric oxide and hydrogen peroxide in heat shock-induced hypericin production of *Hypericum perforatum* suspension cells. Sci. China Ser. C Life Sci..

[14] Sahay S., Gupta M. (2017). An update on nitric oxide and its benign role in plant responses under metal stress. Nitric Oxide.

[15] Clark D., Durner J., Navarre D.A., Klessig D.F. (2000). Nitric oxide inhibition of tobacco catalase and ascorbate peroxidase. Mol. Plant Microbe. Interact..

[16] Murgia I., Tarantino D., Vannini C., Bracale M., Carravieri S., Soave C. (2004). *Arabidopsis thaliana* plants overexpressing thylakoidal ascorbate peroxidase show increased resistance to paraquat-induced photooxidative stress and to nitric oxide-induced cell death. Plant J..

[17] De-Michele R., Vurro E., Rigo C., Costa A., Elviri L., Di-Valentin M., Careri M., Zottini M., Sanita-di-Toppi L., Schiavo F.L. (2009). Nitric oxide is involved in cadmium induced programmed cell death in *Arabidopsis* suspension cultures. Plant Physiol..

[18] Groß F., Durner J., Gaupels F. (2013). Nitric oxide, antioxidants and prooxidants in plant defence responses. Front. Plant Sci..

[19] He H., He L., Gu M. (2014). The diversity of nitric oxide function in plant responses to metal stress. Biometals.

[20] Terrón-Camero L.C., Peláez-Vico M., Del-Val C., Sandalio L.M., Romero-Puertas M.C. (2019). Role of nitric oxide in plant responses to heavy metal stress: Exogenous application versus endogenous production. J. Exp. Bot..

[21] Xiong J., Lu H., Lu K., Duan Y., An L., Zhu C. (2009). Cadmium decreases crown root numbers by decreasing endogenous nitric oxide, which is indispensable for crown root primordial initiation in rice seedlings. Planta.

[22] Kaur G., Singh H.P., Batish D.R., Mahajan P., Kohli R.K., Rishi V. (2015). Exogenous nitric oxide (NO) interferes with lead (Pb)-induced toxicity by detoxifying reactive oxygen species in hydroponically grown wheat (*Triticum aestivum*) roots. PLoS ONE.

[23] Ma W., Xu W., Xu H., Chen Y., He Z., Ma M. (2010). Nitric oxide modulates cadmium influx during cadmium-induced programmed cell death in tobacco BY-2 cells. Planta.

[24] Verma K., Mehta S.K., Shekhawat G.S. (2013). Nitric oxide (NO) counteracts cadmium induced cytotoxic processes mediated by reactive oxygen species (ROS) in *Brassica juncea*: Cross-Talk between ROS, NO and antioxidant responses. Biometals.

[25] Lee B.R., Hwang S. (2015). Over-expression of NtHb1 encoding a non-symbiotic class 1 hemoglobin of tobacco enhances a tolerance to cadmium by decreasing NO (nitric oxide) and Cd levels in *Nicotiana Tabacum*. Environ. Exp. Bot..

[26] Besson-Bard A., Gravot A., Richaud P., Auroy P., Duc C., Gaymard F., Taconnat L., Renou J.P., Pugin A., Wendehenne D. (2009). Nitric oxide contributes to cadmium toxicity in *Arabidopsis* by promoting cadmium accumulation in roots and by up-regulating genes related to iron uptake. Plant Physiol..

[27] Zhao J. (2007). Interplay among nitric oxide and reactive oxygen species a complex network determining cell survival or death. Plant Signal. Behav..

[28] Pietrowska E., Różalska S., Kaźmierczak A., Nawrocka J., Małolepsza U. (2015). Reactive oxygen and nitrogen (ROS and RNS) species generation and cell death in tomato suspension cultures-*Botrytis cinerea* interaction. Protoplasma.

[29] Duan Y.F., Zhang W.S., Li B., Wang Y.N., Li K.X., Han C.Y., Zhang Y.Z., Li X. (2010). An endoplasmic reticulum response pathway mediates programmed cell death of root tip induced by water stress in *Arabidopsis*. New Phytol. Trust.

[30] Liao W., Huang G., Yu J., Zhang M., Shi X. (2011). Nitric oxide and hydrogen peroxide are involved in indole-3-butyric acid-induced adventitious root development in marigold. J. Hortic. Sci. Biotech..

[31] Lum H.K., Butt Y.K., Lo S.C. (2002). Hydrogen peroxide induces a rapid production of nitric oxide in mung bean (*Phaseolus aureus*). Nitric Oxide.

[32] Li L.Z., Yu S.Y., Peijnenburg W.J., Luo Y.M. (2017). Determining the fluxes of ions (Pb^2+^, Cu^2+^ and Cd^2+^) at the root surface of wetland plants using the scanning ion-selective electrode technique. Plant Soil.

[33] McLamore E.S., Porterfield D.M., Banks M.K. (2009). Non-invasive self-referencing electrochemical sensors for quantifying real-time biofilm analyte flux. Biotechnol. Bioeng..

[34] Azmat R., Haider S., Riaz M. (2009). An inverse relation between Pb^2+^ and Ca^2+^ ions accumulation in *phaseolus mungo* and *lens culinaris* under Pb stress. Pak. J. Bot..

[35] Shahid M., Pourrut B., Dumat C., Nadeem M., Aslam M., Pinelli E. (2014). Heavy-metal-induced reactive oxygen species: Phytotoxicity and physicochemical changes in plants. Rev. Environ. Contam. Toxicol..

[36] Shahid M., Pinelli E., Pourrut B., Silvestre J., Dumat C. (2011). Lead-induced genotoxicity to *Vicia faba* L. roots in relation with metal cell uptake and initial speciation. Ecotox. Environ. Safe..

[37] Grover P., Rekhadevi P.V., Danadevi K., Vuyyuri S.B., Mahboob M., Rahman M.F. (2010). Genotoxicity evaluation in workers occupationally exposed to lead. Int. J. Hyg. Envirn. Health.

[38] Gupta D.K., Nicoloso F.T., Schetinger M.R., Rossato L.V., Huang H.G., Srivastava S., Yang X.E. (2011). Lead induced responses of *Pfaffia glomerata*, an economically important Brazilian medicinal plant, under in vitro culture conditions. Bull. Environ. Contam. Toxicol..

[39] Kopittke P.M., Asher C.J., Blamey F.P.C., Menzies N.W. (2007). Toxic effects of Pb^2+^ on the growth and mineral nutrition of signal grass (*Brachiaria decumbens*) and Rhodes grass (*Chloris gayana*). Plant Soil.

[40] Mourato M., Moreira I., Leitão I., Pinto F., Sales J., Martins L. (2015). Effect of heavy metals in plants of the genus *Brassica*. Int. J. Mol. Sci..

[41] Kobylińska A., Posmyk M.M. (2016). Melatonin restricts Pb-induced PCD by enhancing BI-1 expression in tobacco suspension cells. Biometals.

[42] Reddy A.M., Kumar S.G., Jyothsnakumari G., Thimmanaik S., Sudhakar C. (2005). Lead induced changes in antioxidant metabolism of horsegram (*Macrotyloma uniflorum* (Lam.) Verdc.) and bengalgram (*Cicer arietinum* L.). Chemosphere.

[43] Verma S., Dubey R.S. (2003). Lead toxicity induces lipid peroxidation and alters the activities of antioxidant enzymes in growing rice plants. Plant Sci..

[44] Singh H.P., Kaur S., Batish D.R., Sharma V.P., Sharma N., Kohli R.K. (2009). Nitric oxide alleviates arsenic toxicity by reducing oxidative damage in the roots of *Oryza sativa* (rice). Nitric Oxide.

[45] Yu C.C., Hung K.T., Kao C.H. (2005). Nitric oxide reduces Cu toxicity and Cu-induced NH^4+^ accumulation in rice leaves. J. Plant Physiol..

[46] Singh H.P., Batish D.R., Kaur G., Arora K., Kohli R.K. (2008). Nitric oxide (as sodium nitroprusside) supplementation ameliorates Cd toxicity in hydroponically grown wheat roots. Environ. Exp. Bot..

[47] Lindermayr C., Durner J. (2015). Interplay of reactive oxygen species and nitric oxide: Nitric oxide coordinates reactive oxygen species homeostasis. Plant Physiol..

[48] Xu J., Yin H.X., Li Y.L., Liu X.J. (2010). Nitric oxide is associated with long-term zinc tolerance in *Solanum nigrum*. Plant Physiol..

[49] Arora D., Jain P., Singh N., Kaur H., Bhatla S.C. (2016). Mechanisms of nitric oxide crosstalk with reactive oxygen species scavenging enzymes during abiotic stress tolerance in plants. Free Radic. Res..

[50] Gill S.S., Hasanuzzaman M., Nahar K., Macovei A., Tuteja N. (2013). Importance of nitric oxide in cadmium stress tolerance in crop plants. Plant Physiol. Biochem..

[51] Pető A., Lehotai N., Feigl G., Tugyi N., Ördög A., Gémes K., Tari I., Erdei L., Kolbert Z. (2013). Nitric oxide contributes to copper tolerance by influencing ROS metabolism in *Arabidopsis*. Plant Cell Rep..

[52] Sadeghipour O. (2016). Pretreatment with nitric oxide reduces lead toxicity in cowpea (*Vigna unguiculata* [L.] walp.). Arch. Biol. Sci..

[53] Huang Y., Cao H., Yang L., Chen C., Shabala L., Xiong M., Niu M., Liu J., Zheng Z., Zhou L. (2019). Tissue-specific respiratory burst oxidase homologue-dependent H_2_O_2_ signaling to the plasma membrane H^+^-ATPase confers potassium uptake and salinity tolerance in Cucurbitaceae. J. Exp. Bot..

[54] Fang K.F., Du B.S., Zhang Q., Xing Y., Cao Q.Q., Qin L. (2019). Boron deficiency alters cytosolic Ca^2+^ concentration and affects the cell wall components of pollen tubes in *Malus domestica*. Plant Biol..

[55] Lamhamdi M., El-Galiou O., Bakrim A., Nóvoa-Muñoz J.C., Arias-Estevez M., Aarab A., Lafont R. (2013). Effect of lead stress on mineral content and growth of wheat (*Triticum aestivum*) and spinach (*Spinacia oleracea*) seedlings. Saudi. J. Biol. Sci..

[56] Zhou J., Fan C., Liu K., Jing Y. (2015). Extracellular ATP is involved in the initiation of pollen germination and tube growth in *Picea meyeri*. Trees.

