# Supplementary material for: Nitric Oxide Enhances Cytotoxicity of Lead by Modulating the Generation of Reactive Oxygen Species and Is Involved in the Regulation of Pb2+ and Ca2+ Fluxes in Tobacco BY-2 Cells
Source: Plants (Basel). 2019 Oct 9;8(10):403. doi: 10.3390/plants8100403 (PMC6843202; doi:10.3390/plants8100403)
Supplement: Supplementary file 1 [file plants-08-00403-s001.pdf]

Supplementary Materials

Supplementary Method

TUNEL Assay

The PCD of tobacco BY-2 cells was determined with a TUNEL apoptosis detection kit (DeadEnd™ Fluorometric TUNEL System, Promega, Madison, WI, USA) according to the manufacturer's instructions. Briefly, cells were washed in PBS, and 50 μL of the BY-2 cells were pipetted onto poly-L-lysine-coated slides. Then, the cells were fixed by immersing the slides in freshly prepared 4% paraformaldehyde solution in PBS for 30 min at 4 °C and incubated in 50 μL of the TUNEL reaction mixture for 1 h in the dark at 37 °C. After that, the samples were stained with PI (1 μg mL^{-1}) for 15 min at room temperature in the dark. A negative control was carried out without terminal deoxynucleotidyl transferase (TdT) and a positive control was carried out with DNase I before the TUNEL reaction. The fluorescence of the samples was detected using a fluorescence microscope (Olympus BX61, Tokyo, Japan).

Supplementary Figures

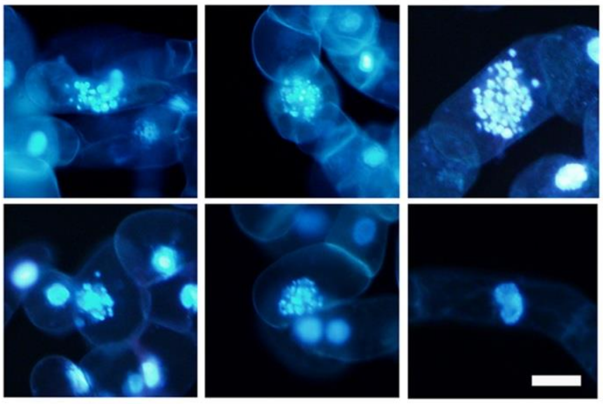

Figure S1. Typical cells with nuclei containing condensed or granular chromatin. Hoechst 33342 staining in cultured tobacco BY-2 cells treated with 250 μM $Pb(NO_3)_2$ for 24 h. Scale bar = 50 μm.

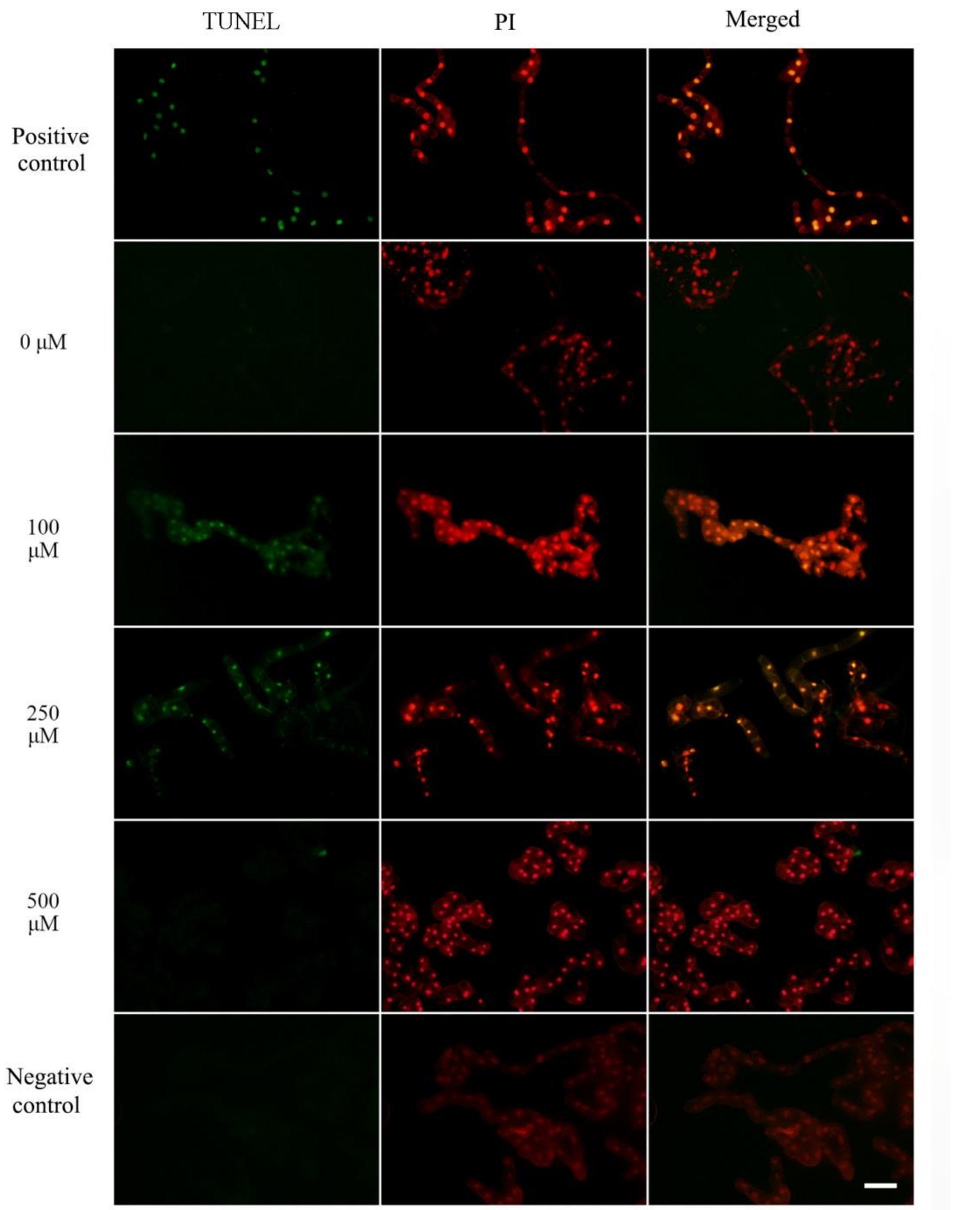

Figure S2. Programmed cell death detection using the TUNEL assay. Tobacco BY-2 cells that received the same volume of distilled water were used as a control. Four-day-old tobacco BY-2 cells were treated with different concentrations (100, 250, and 500 µM) of $Pb(NO_3)_2$ for 24 h. Left column: TUNEL images; middle column: PI images; right column: merged images of TUNEL and PI. Scale bar = 100 µm.
